# Phenotypic and Functional Characterization of Müller Glia Isolated from Induced Pluripotent Stem Cell‐Derived Retinal Organoids: Improvement of Retinal Ganglion Cell Function upon Transplantation

**DOI:** 10.1002/sctm.18-0263

**Published:** 2019-04-29

**Authors:** Karen Eastlake, Weixin Wang, Hari Jayaram, Celia Murray‐Dunning, Amanda J. F. Carr, Conor M. Ramsden, Anthony Vugler, Katrina Gore, Nadine Clemo, Mark Stewart, Pete Coffey, Peng T. Khaw, G. Astrid Limb

**Affiliations:** ^1^ NIHR Biomedical Research Centre UCL Institute of Ophthalmology and Moorfields Eye Hospital London United Kingdom; ^2^ Apollo Therapeutics Stevenage United Kingdom

**Keywords:** Stem cells, Induced pluripotent stem cell, Müller glia, Glaucoma, Regeneration

## Abstract

Glaucoma is one of the leading causes of blindness, and there is an ongoing need for new therapies. Recent studies indicate that cell transplantation using Müller glia may be beneficial, but there is a need for novel sources of cells to provide therapeutic benefit. In this study, we have isolated Müller glia from retinal organoids formed by human induced pluripotent stem cells (hiPSCs) in vitro and have shown their ability to partially restore visual function in rats depleted of retinal ganglion cells by NMDA. Based on the present results, we suggest that Müller glia derived from retinal organoids formed by hiPSC may provide an attractive source of cells for human retinal therapies, to prevent and treat vision loss caused by retinal degenerative conditions. stem cells translational medicine *2019;8:775&784*

**Significance Statement**

There is a need for novel therapies to treat retinal degenerative conditions such as glaucoma. The authors suggest that Müller cells isolated from induced pluripotent stem cells (iPSCs)‐derived retinal organoids may constitute a well‐traceable source of cells to develop such therapies. The study shows that intravitreal transplantation of iPSC‐derived Müller glia into an experimental rat model of retinal ganglion cell depletion can partially restore visual function. This response was judged by an improvement of the negative scotopic threshold response of the electroretinogram. The results suggest that iPSC‐derived Müller glia constitute an important source of cells for human retinal therapies.

## Introduction

Glaucoma is one of the leading causes of blindness throughout the world 1. It is characterized by high intraocular pressure, gradual loss of retinal ganglion cells (RGCs), and optic nerve damage 2, 3. Current strategies to treat glaucoma only slow progression of the disease, and not all patients respond well to treatment, leading to severe sight loss and visual disability. Recent studies indicate that cell transplantation therapies may be developed with the aim to provide neurotrophic support to maintain the viability and function of remaining neurons and to potentially repair axonal damage.

Müller glia with stem cell characteristics were first identified in the zebrafish 4, in which they are responsible for the complete regeneration of the adult retina after injury 5, 6. In this species, Müller glia re‐enter the cell cycle to generate multipotent progenitors that proliferate, migrate, and differentiate into most neural cell types 7, that also restore retina function 8. Although complete retinal regeneration has not been observed in other species, limited regenerative potential of Müller glia has been observed in chick 9 and rodent 10, 11 retinae. In rodent retina in vivo, it is reported that Müller glia can re‐enter the mitotic cycle to generate amacrine cells in response to growth factors 10 or photoreceptors in response to N‐methyl‐D‐aspartate (NMDA) 11. A population of Müller glia isolated from the adult human retina has also been shown to have stem cell characteristics (human Müller stem cells [hMSC]) in vitro. These cells, can be isolated from cadaveric donors, become spontaneously immortalized in vitro, and acquire markers and function of retinal neurons after culture with various growth and differentiation factors 12, 13, 14. However, there is no evidence of regeneration occurring after disease or injury in humans. That Müller glia may have potential for therapeutic application in glaucoma derives from experimental studies showing that hMSCs have the ability to partially restore visual function in rodent and feline models of NMDA‐induced RGC damage 15, 16. In addition, when directed toward a photoreceptor fate, these cells were shown to improve rod function in the P2H3 rat (a model of retinitis pigmentosa) after subretinal transplantation 17. Müller glia derived from cadaveric donors present major difficulties for clinical application because of the risks of disease transmission caused by prions and nonidentified pathogens, as well as limitations because of the histocompatibility issues. Pluripotent stem cells, however, have the potential to overcome these issues, and recent studies have shown that retinal organoids that exhibit the characteristics of a whole laminated neural retina can be generated by both human induced pluripotent stem cells (hiPSC) and embryonic stem cells (hESC) in vitro 18, 19. Various sources of hESCs and hiPSCs that comply with regulatory requirements for human therapies have become available and this will potentially facilitate the derivation of cells for retinal therapies in the near future.

This study aimed to isolate Müller glia from retinal organoids formed by hiPSC cells in vitro and to assess their ability to partially restore visual function in rats depleted of RGC by NMDA. We examined the purity and nature of the cells isolated from these organoids by determining the gene and protein expression of well‐known markers of Müller glia. We also investigated the ability of these cells to partially restore visual function upon transplantation into the vitreous of rats depleted of RGC as previously shown with Müller glia isolated from adult human retina 15. The outcome of the transplantation was evaluated by assessing the negative scotopic threshold response (nSTR) of the electroretinogram (ERG) and by immunohistochemical analysis of the transplanted retina in vitro. Based on the present results, we propose that Müller glia derived from retinal organoids formed by hiPSC may provide an attractive source of cells for human retinal therapies, to prevent and repair vision loss caused by retinal degenerative conditions such as glaucoma.

## Materials and Methods

### Stem Cell Maintenance and Retinal Organoid Differentiation

**Figure 1:**
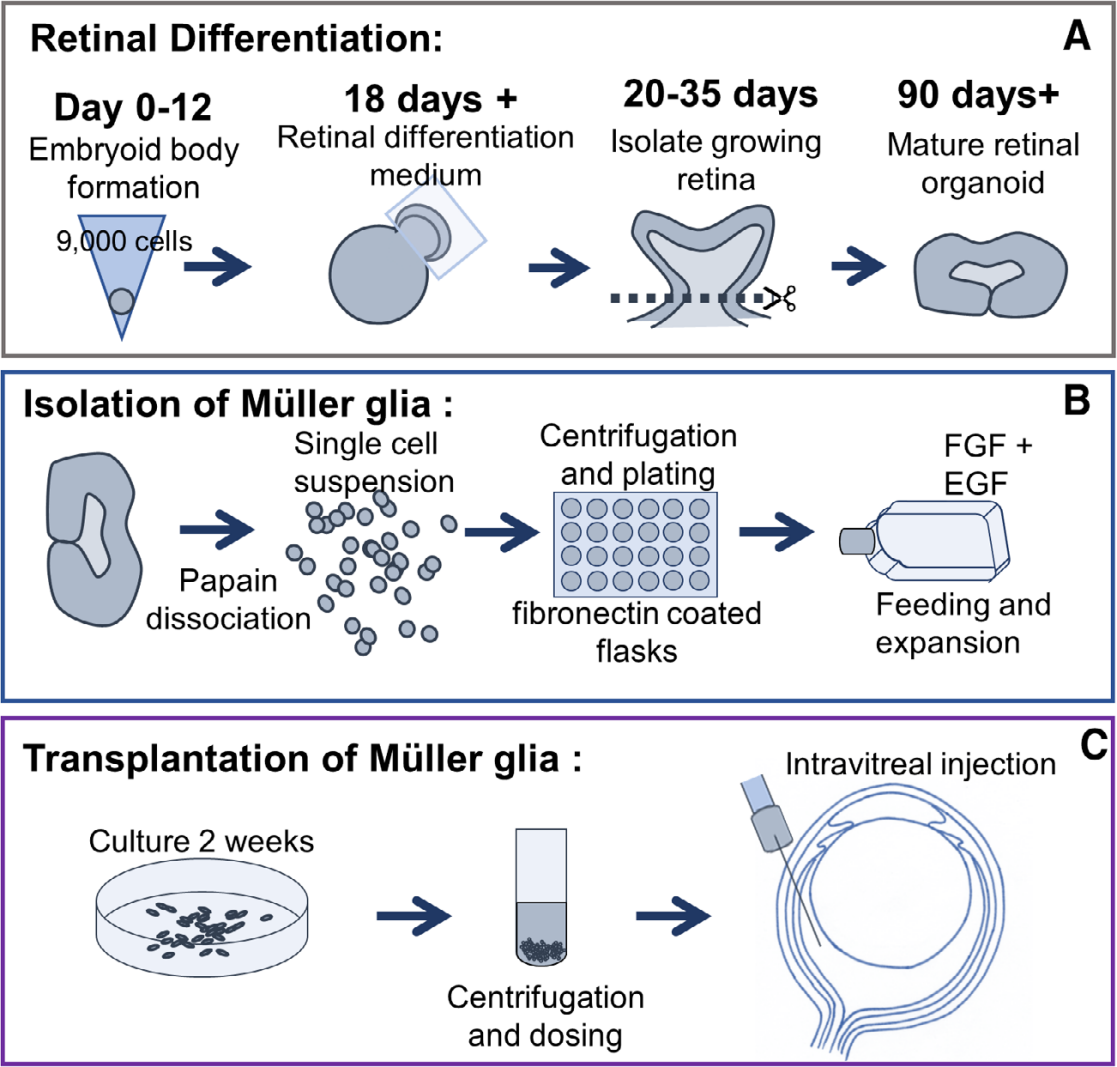
Schematic summary representation of the methods used in retinal organoid differentiation and Müller glia isolation for transplantation. **(A):** Formation of retinal organoids from induced pluripotent stem cells, from embryoid body formation to isolation of the growing retina. **(B):** Retinal organoids were dissociated using papain to obtain a single cell suspension. **(C):** Isolated Müller glia from retinal organoids were grown into confluent monolayers before harvest for intravitreal injection into the rat eye.

The hiPSC line used in this study was created from BJ fibroblasts (Stemgent, Glasgow, U.K. Cat. No. 08‐0027) as previously described by Carter et al. 20. BJ iPSC cells were maintained in TeSR‐E8 (StemCell Technologies, Cambridge, U.K.) and grown on Matrigel (Corning, St David's Park, U.K.)‐coated six‐well plates. Differentiation of human pluripotent stem cells into retinal organoids was based on previous protocols developed by Nakano et al. 18 and Parfitt et al. 21. Upon confluence, cells were washed with ×1 phosphate‐buffered saline (PBS) and dissociated with TryplE (ThermoFisher, U.K.) containing 10 μM ROCKi (Y‐27632, Millipore, Watford, U.K.) and 0.5 mg/ml DNase (Sigma‐Aldrich, U.K.) and pelleted. Cells were seeded to a density of 9,000 cells per well in a v‐bottomed 96‐well plate (PrimeSurface Sumilon low adhesion, Alpha Laboratories, U.K.) in Glasgow Minimum Essential Medium (GMEM) with l‐glutamine containing 20% KOSR (ThermoFisher), 1% ×100 sodium pyruvate (ThermoFisher, U.K.), 1% ×100 nonessential amino acids (ThermoFisher), 1% ×100 penicillin/streptomycin (ThermoFisher, U.K.), and 50 μM β‐mercaptoethanol (ThermoFisher, U.K.) containing 20 μM ROCKi and 3 μM Wnt antagonist (Millipore, Watford, U.K.). After 2 days in culture, wells were topped up with 100 μl of GMEM media containing 20% Knockout serum replacement (KOSR), 1% ×100 sodium pyruvate, 1% ×100 nonessential amino acids, 1% ×100 penicillin/streptomycin, 50 μM β‐mercaptoethanol, 20 μM ROCKi, 3 μM Wnt antagonist, and 2% Matrigel. Half media change was performed twice weekly. On day 12, embryoid bodies (EBs) formed were transferred into separate wells of a 25‐well squared, low‐adhesion plate, and incubated in GMEM with l‐glutamine, 20% KOSR, 10% fetal calf serum (FCS) 1% ×100 sodium pyruvate, 1% ×100 nonessential amino acids, 1% ×100 penicillin/streptomycin, and 50 μM β‐mercaptoethanol containing 1% Matrigel and 100 nM smoothened agonist (SAG; Millipore, Watford, U.K.). On day 15, the same media was replaced containing 1% Matrigel and 100 nM SAG. On day 18, medium was replaced with Dulbecco's modified Eagle's medium/F12‐glutamax containing 10% FCS, 1% ×100 N2 supplement (ThermoFisher, U.K.; 1% penicillin/streptomycin/amphotericin and 0.5 μM retinoic acid [Sigma‐Aldrich, U.K.]). EBs, from which retinal organoids become visible between days 18 and 30, were then fed twice weekly. From days 30 to 50, retinal organoids were cut from the EBs under the microscope using microblades and placed into new 25 square low‐adhesion plates. This was to ensure that only neural retinal tissue remained and matured in culture. Before dissociation, any pigmented cell, occasionally forming with the organoids, was removed to prevent any possible retinal pigment epithelial contamination in the growing cultures. For long term, culture medium was replaced twice weekly (illustrated in Fig. 1A). All cells were maintained at 37°C, 5% CO_2_, and atmospheric O_2._

### Isolation of Müller Glia from Retinal Organoids

Müller cells were harvested from retinal organoids between 30 and 281 days after initiation of retinal differentiation for characterization analysis. Cells used for intravitreal transplantations were derived from organoids at day 281. Organoids were dissociated using a papain dissociation kit protocol supplied by the manufacturer (Worthington Biochemical, Lakewood, NJ, USA). Briefly, retinal organoids were incubated with papain for approximately 10‐15 minutes at 37°C with agitation by pipetting every 5 minutes. Pelleted cell suspensions were then plated as described previously for Müller glia isolated from human cadaveric retina (illustrated in Fig. 1B).

### Immunohistochemistry of Retinal Organoids and Isolated Cells

Cells were grown to a confluent monolayer on fibronectin‐coated glass coverslips and washed in PBS before fixation in 4% paraformaldehyde for 5 minutes. After fixation, cells were cryoprotected in 30% sucrose for 30 minutes and allowed to dry before freezing. For use, cells were defrosted and 500 μl of tris‐buffered saline (TBS) + 0.3% triton X was added to the cells. Cells were blocked for 1 hour in TBS + 0.3% triton + 5% donkey serum before the addition of the primary antibody (diluted in blocking buffer). Primary antibodies (see Supporting Information Table S1) were incubated overnight at 4°C. Cells were then washed with TBS three times for 5 minutes. Secondary antibodies (Alexa flour, Invitrogen, U.K. 1:500 in TBS + 0.3% triton) were incubated for 3 hours at room temperature in the dark. Slides were then washed in TBS and coverslips mounted with Fluoroshield Mounting Medium containing 4′,6‐diamidino‐2‐phenylindole (DAPI; Abcam, Cambridge, U.K.), sealing with nail varnish.

### Flow Cytometry Analysis

Cells were detached from tissue culture flasks to obtain a single cell suspension. These were then washed in PBS to remove any remaining tissue culture medium. Cells were then incubated with primary antibodies to CD29, CD44 (positive controls), SSEA4, and cytokeratin‐18 (negative control markers) for 30 minutes in the dark. The cells were then washed three times by centrifugation and resuspended in sterile PBS. Corresponding isotype controls and a no primary antibody cell suspension were used as negative controls (see Supporting Information Table S1). Fluorescence‐activated cell sorting (FACS) analysis was performed using a The BD LSRFortessa X‐20 cell analyzer (BD Biosciences, Berkshire, U.K.). Supporting Information Fig. S1 shows data for negative markers SSEA4 and cytokeratin‐18, and Supporting Information Fig. S2 shows flow cytometry data for all isotype controls.

### Gene Expression Analysis by Reverse Transcription‐Polymerase Chain Reaction

Total cellular RNA was isolated from cell pellets using the RNeasy system (Qiagen, Hilden, Germany; http://www.qiagen.com). For each reverse transcription reaction, 1 μg of total RNA was reverse transcribed in 20‐μl reactions by adding 1 μl of oligo d(T)12‐18 primers (Invitrogen) and 1 μl of dNTP (Promega) to each sample and incubated for 5 minutes at 65°C in a thermal cycler before adding 4 μl of first strand buffer (Invitrogen), 1 μl 0.1 M DTT, 0.5 μl Rnase inhibitor (RNasin Plus, N2611, Promega, U.K.), and 1 μl of SuperScript IV (Invitrogen) and incubating in the thermal cycler for a further 10 minutes at 55°C and 10 minutes at 80°C. Polymerase chain reaction (PCR) amplification was performed in 20‐μl volume by addition of 10 μl of GoTaq Green master mix (Cat. No. M712; Promega, U.K.), which contains a mix Taq DNA polymerase, dNTPs, MgCl2, and reaction buffers and was used along with 7 μl RNase free water, 1 μl of each forward and reverse primers, and 1 μl cDNA. The mixture was incubated at 94°C for 5 minutes, followed by an appropriate number of cycles as follows: 94°C for 1 minute, annealing temperature °C for 1 minute, 72°C for 1 minute; and one cycle of 72°C for 5 minutes. PCR products were analyzed by agarose gel electrophoresis (2%) containing gel red (Supporting Information Table S2) for primer sequences.

### Animal Husbandry, Tolerization, Immunosuppression, and Anesthesia

Wild‐type Lister hooded rats were maintained according to the U.K. Home Office regulations for the care and use of laboratory animals (Scientific Procedures Act 1986). The use of the animal species for the study was approved by the local ethics committee at University College London, Institute of Ophthalmology and the U.K. Home Office. The animals were given access to food and water ad libitum and kept under 12‐hour light/12‐hour dark cycles. All animals used in the study were tolerized within 24 hours of birth by an intraperitoneal injection of 1 × 10^5^ Müller glia derived from iPSC retinal organoids using protocols described previously 15. Anesthetic for intravitreal transplantation procedures consisted of ketamine (60 mg/kg) and xylazine (7.5 mg/kg). Immunosuppression, started 2 days before cell transplantations, was administered daily in drinking water, and consisted of 25 mg azathioprine, 5 mg prednisolone, and 210 mg cyclosporin per liter.

### Induction of RGC Damage by NMDA and Intravitreal Müller Cell Transplantation

Four‐week‐old animals were anesthetized and pupils treated with tropicamide (1% w/v, Minims; Bausch and Lomb, Kingston‐upon‐Thames, Surrey, U.K.), phenylephrine hydrochloride (2.5% w/v, Minims; Bausch and Lomb, Kingston‐upon‐Thames, Surrey, U.K.), and oxybuprocaine hydrochloride (0.4% w/v; Minims Bausch and Lomb, Kingston‐upon‐Thames, Surrey, U.K.) drops. Viscotears (Bausch and Lomb, Kingston‐upon‐Thames, Surrey, U.K.) were used to make sure the fellow eye did not dry out during the procedure. Injections were performed using a 5 μl Hamilton syringe and 32G needle. RGC damage was induced by injection of 2 μl of a mixture of NMDA (80 μM) and triamcinolone (80 mg/ml) into the intravitreal space of the left eye. To allow for assessment of RGC damage at 1 week after NMDA injection (Supporting Information Fig. S3), we performed ERG analysis at this time point. To comply with U.K. Home Office regulations and to allow for anesthetic recovery from the ERG test, cells were transplanted a week after the first ERG test. Animals were injected with 2 μl of a cell suspension consisting of 1 μl chondroitinase ABC and 1 μl of cells into the same eye (illustrated in Fig. 1C). Because Müller cells were frozen in small numbers upon isolation from the organoids, to obtain sufficient number of Müller glia for transplantation and characterization, we expanded these cells over a period of 2 weeks before injection. Three different injections were given to each of three experimental groups (*n* = 16 for each group) as follows—group A: medium‐no cells (sham); group B: 4 × 10^4^ Müller glial cells derived from hiPSC retinal organoids; and group C: 1 × 10^5^ cells Müller glial cells derived from hiPSC retinal organoids. Four weeks after transplantation, animals were assessed for their RGC function by dark adapted ERGs and subsequently euthanized by terminal anesthesia, followed by paraformaldehyde (4%) perfusion.

### Scotopic ERG Recordings

Animals were dark adapted overnight before ERGs. Rats were anesthetized as described above, and both eyes treated with tropicamide (1% w/v) and phenylephrine hydrochloride (2.5% w/v) to dilate the pupils and oxybuprocaine hydrochloride (0.4% w/v) to anesthetize. Viscotears were used to ensure the cornea remained hydrated. Animals were housed in a Faraday cage for ERG recordings and placed on a heated table to control the body temperature, in a Ganzfeld stimulator (Colordome; Diagnosys, Cambridge, U.K.; http://diagnosysllc.com). Flash stimuli (4‐millisecond duration, repetition rate of 0.13 Hz) were presented in the Ganzfeld color dome by light‐emitting diode stimulator at intensities of −6.5 to −2 log cd second/m^2^. The flash stimulus was repeated 30 times for each light intensity. The responses were measured using the Espion Diagnosys V6 software. Measurements for nSTR analysis were analyzed by determination and recording of the maximal negative response in the range of 160–230 milliseconds (for nSTR) at each light intensity.

All ERG analyses were performed on R version 3.4.0 (The R Foundation for Statistical Computing) using the R program “Analyse summary statistics R.” An analysis of variance model was fitted to each intensity in turn. That model investigated effects of day and treatment group. The equivalent ERG summary post‐NMDA was considered as a potential covariate but no relationship was observed (*p* > .10), so the summary was not included in the model. Least square (LS) means were obtained for each treatment group, and differences in mean response to the control group were presented together with corresponding 95% confidence intervals (CI). As an additional exploration, a mixed effects model was fitted to data for intensities −4.5 to −3 combined. The fixed effects included in the model were day, intensity, treatment, and treatment by intensity interaction. Animal was fitted as a random effect. The LS means were obtained as an averaged for each treatment across the intensities used in the test, and differences in average response to the control group were presented, together with 95% CI. The assumptions of all models were assessed visually using diagnostic plots.

### Immunocytochemistry and Confocal Microscopy for Visualizing Transplanted Cells

After perfusion fixation, eyes were enucleated and placed in 4% paraformaldehyde overnight before replacing with 30% sucrose for cryopreservation. The lens was then removed from each eye and the remaining orbit embedded in OCT before sectioning at 16 μM using a Leica cryostat and mounting on Superfrost plus slides (VWR, U.K.). Sections were washed with TBS and blocked at room temperature with TBS containing 0.3% triton and 5% donkey serum for 1 hour. To identify the transplanted human Müller glia in the rat eyes, mouse monoclonal antibodies to human CD29 (1:300; Santa Cruz Biotechnology, U.S.A) and human Nestin (1:300; Millipore, Watford, U.K.) were used in conjunction. Primary antibodies were diluted in blocking buffer and incubated overnight at 4°C. Slides were then washed 3 times in TBS, and both primary antibodies were probed with the secondary antibody Alexa fluor 488 (Invitrogen, U.K.) (1:500; 3 hours; Thermo Fischer, U.K.). Slides were washed in TBS 3 times before addition of a mounting media containing DAPI, covered with a glass coverslip, and sealed with nail varnish for microscope examination. Immunofluorescence staining was captured using a Leica 710 confocal microscope.

## Results

### Müller Glia Development Within Retinal Organoids

**Figure 2:**
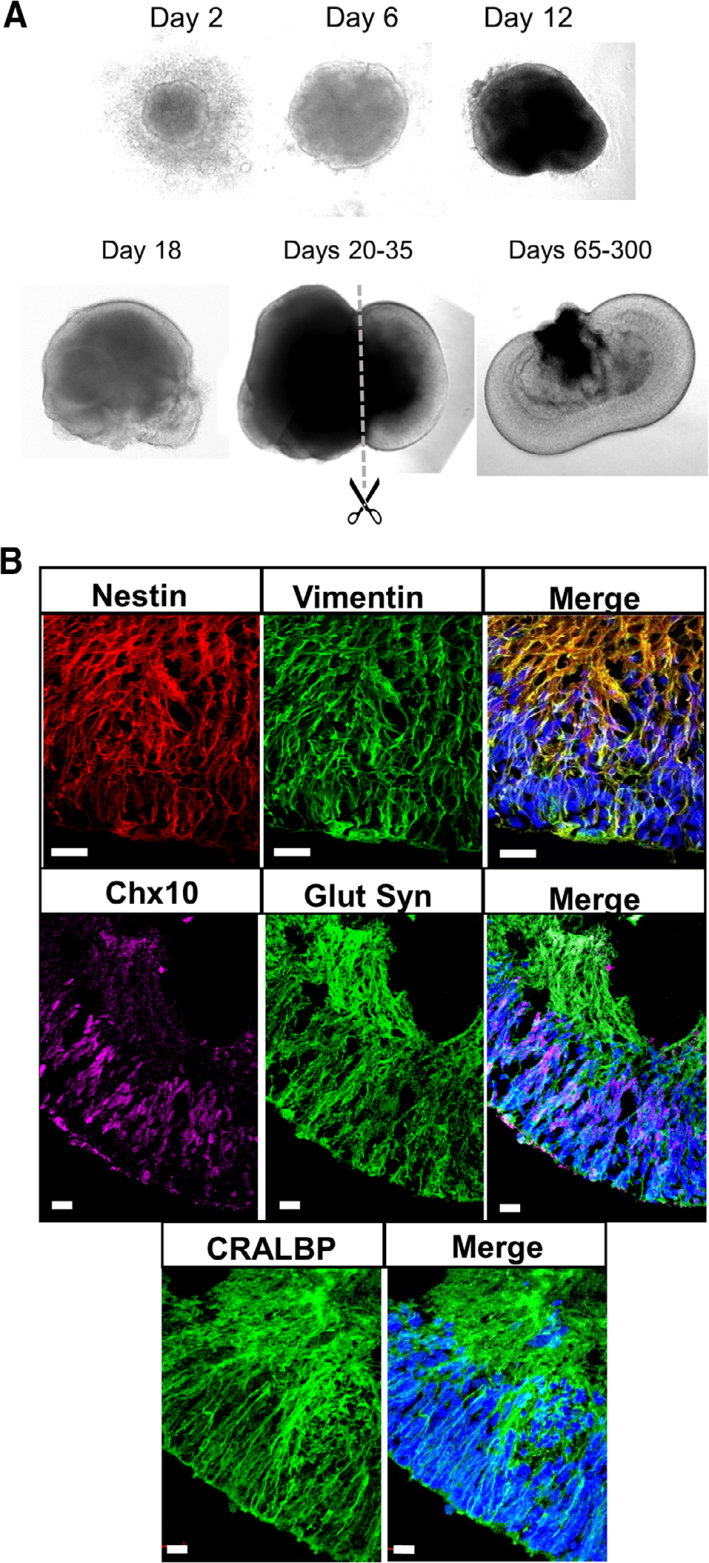
Retinal organoid development and Müller glia identification. **(A):** Representative phase microscopy images of retinal organoid development from induced pluripotent stem cells from days 2 to 300. **(B):** Confocal microscopy images of a retinal organoid at day 99 after initiation of differentiation, showing expression of the Müller glia‐specific markers CRALBP, nestin, vimentin, Chx10, and glutamine synthetase. Scale bar = 20 μm.

Two days after initiation of the differentiation process, hiPSCs formed cellular spheroids characteristic of EBs. These EBs continued to enlarge, and by day 12, small cell protrusions were seen emerging from these structures. After 18 days, these protrusions were observed expanding on the periphery of the EBs to form nearly transparent cell structures, which denote retinal development. Between days 20 and 35, these retinal structures were sectioned from the EB and transferred to new plates to allow maturation for up to 300 days. During this period, retinal laminations could be increasingly observed by phase contrast microscopy (Fig. 2A). Immunocytochemical staining of these organoids showed that after 90 days from initiation of the retinal differentiation process, Müller glia markers including Nestin, vimentin, glutamine synthetase, Chx10, and Cellular retinaldehyde binding protein (CRALBP) were expressed in cells that extend across the whole width of the retinal structure, which is one of the main features of Müller glia in the mature mammalian retina in vivo (Fig. 2B).

### Characterization of Müller Glia Isolated from Retinal Organoids Formed by iPSC

**Figure 3:**
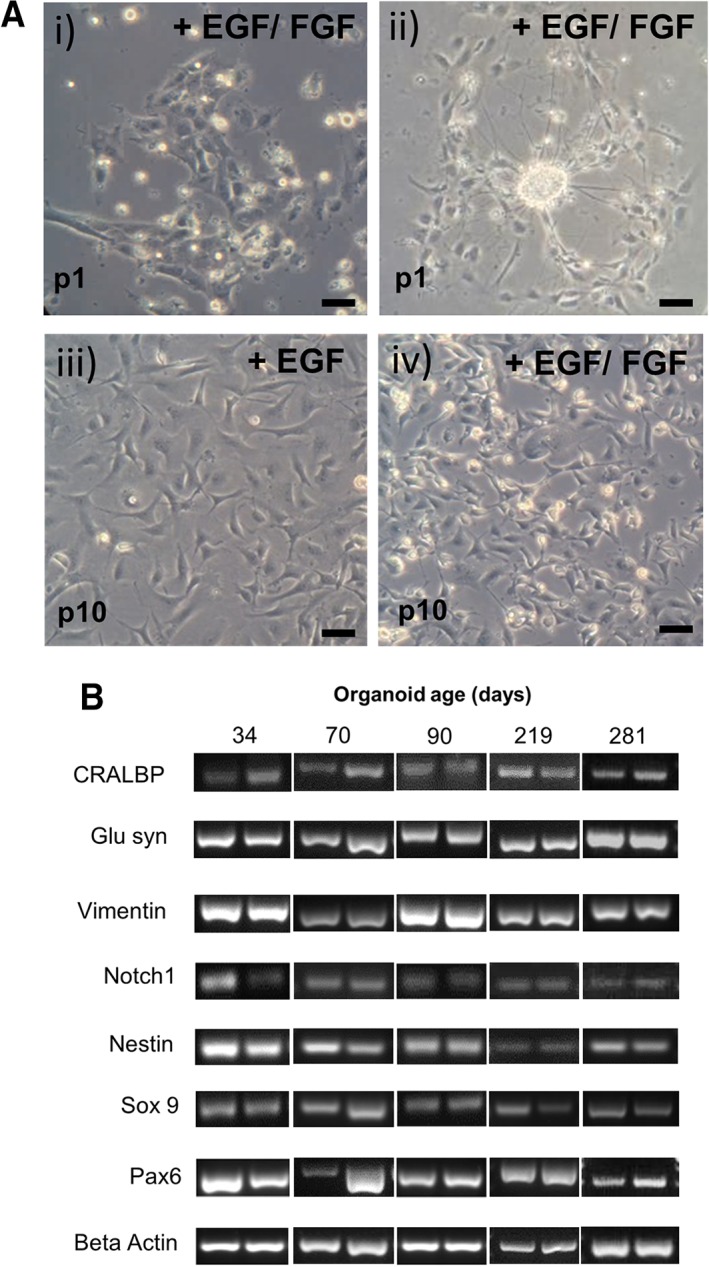
Morphology and expression profile of Müller glia isolated from induced pluripotent stem cell‐derived retinal organoids. **(A):** (i) Appearance of Müller glia after 1 day in culture (ii) characteristic formation of sphere‐like structures at early passages. (iii) Muller glia grown in the presence of EGF alone display typical bipolar morphology and adhesion to culture plates. (iv) Müller glia cultured in the presence of EGF and FGF show fast growth and division as indicated by the presence of phase bright nuclei, characteristic of dividing cells. Scale bar = 100 μm. **(B):** Gel bands represent the expression of mRNA coding for Müller glia and progenitor factors in cells isolated from retinal organoids at different times after initiation of retinal differentiation. Duplicate bands represent two consecutive passages of the same cell preparation.

After dissociation of retinal organoids, cells were plated on fibronectin‐coated culture plates. Initially they formed small cell clusters (Fig. 3Ai) and after a few days in culture, many of these cell groups form neurosphere‐like structures, which is often observed with Müller glia isolated from the adult human retina 13 (Fig. 3Aii). Cells were expanded in the presence of Epidermal growth factor (EGF) and Basic fibroblast growth factor (bFGF), and after 4–6 weeks culture, they were examined for gene and protein expression of Müller glial cell markers. We observed that bFGF greatly increased expansion rate of the cell colonies but was not necessary for continued expansion after the first three passages (Fig. 3Aiii–iv). Proliferating cells showed a characteristic Müller glia bipolar‐like morphology with membrane protrusions, characteristic of Müller glia. This morphology was maintained throughout several passages. If cells were maintained in the presence of FGF for a long term, a morphology characteristic of neurosphere‐like forming cells with shorter processes and phase bright nuclei was often observed (Fig. 3Aiv).

Cells isolated from retinal organoids at 34, 70, 90, 219, and 281 days after initiation of organoid differentiation were examined for the expression of genes coding for Müller glia and neural progenitor markers, the characteristics of adult human Müller glia 13. Expression of mRNA coding for the Müller glia markers CRALBP, glutamine synthetase, nestin, and vimentin was observed in two different passages of all cell preparations (Fig. 3B). In addition, the expression of mRNA coding for the progenitor markers Sox9, Pax6, and notch1 was also observed in two different passages of all cell preparations (Fig. 3B).

### Purity of the Müller Cell Preparation Obtained from Retinal Organoids

**Figure 4:**
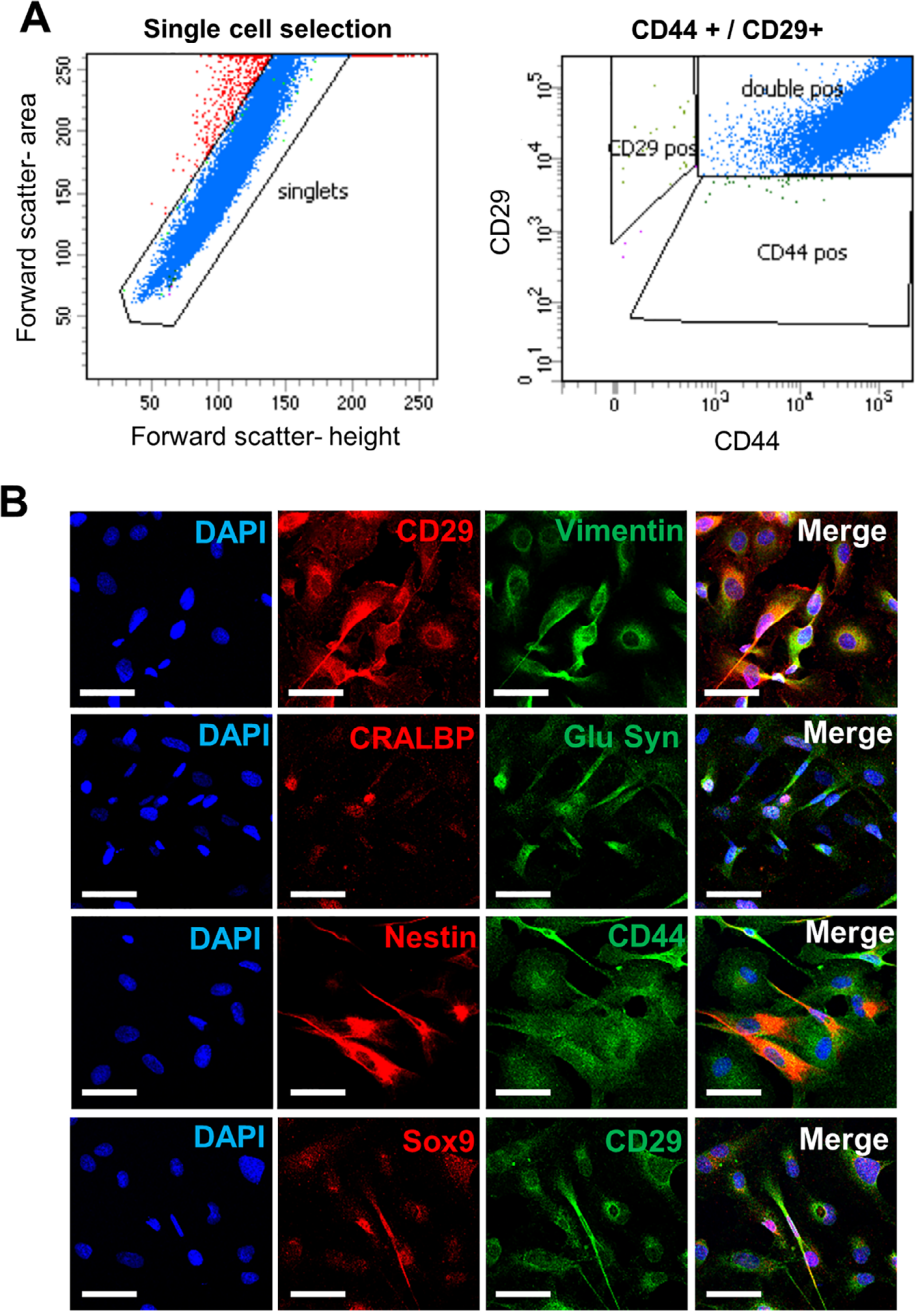
Protein expression profile of Müller glia isolated from induced pluripotent stem cell‐derived retinal organoids. **(A):** Flow cytometry analysis of Müller glia isolated from retinal organoids showing double positive staining for CD44 and CD29 by the majority of cells (>99.7%). **(B):** Immunofluorescence staining of isolated Müller glia cells showing expression of CD29, vimentin, CRALBP, glutamine synthetase, nestin, CD44, and Sox9, characteristic markers of Müller glia. Scale bar = 50 μm. Abbreviation: DAPI, 4′,6‐diamidino‐2‐phenylindole.

Cells isolated from retinal organoids were expanded on fibronectin‐coated culture plates to obtain sufficient number for characterization studies. As determined by flow cytometry (FACS) analysis, the proportion of cells coexpressing the Müller cell surface markers CD44 and CD29 was above 99.7% (Fig. 4A). To confirm specificity, the cell preparations were also examined for the expression of negative markers including SSEA4, a known stem cell marker and cytokeratin‐18, an epithelial cell marker. Both markers were shown to be under 1% positive for our Müller glia cell preparation (Supporting Information Fig. S1). Immunofluorescence staining confirmed the expression of progenitor and Müller glia markers by the isolated retinal cells. Costaining for the cell surface marker CD29 and the intermediate filament protein vimentin showed that all cells were expressing both markers. Well‐known Müller glia markers including CRALBP and glutamine synthetase also showed prominent cytoplasmic staining in the majority of the cells (Fig. 4B). Immunostaining for the Müller glia cell surface marker CD44 was also observed in all the cells, with a large proportion also staining for nestin (Fig. 4B). In addition, nuclear staining for the Muller progenitor marker Sox9 was observed in all cells (Fig. 4B).

### Intravitreal Transplantation of hiPSC Derived Müller Glia Partially Restores RGC Function in a Rat Model of RGC Depletion

**Figure 5:**
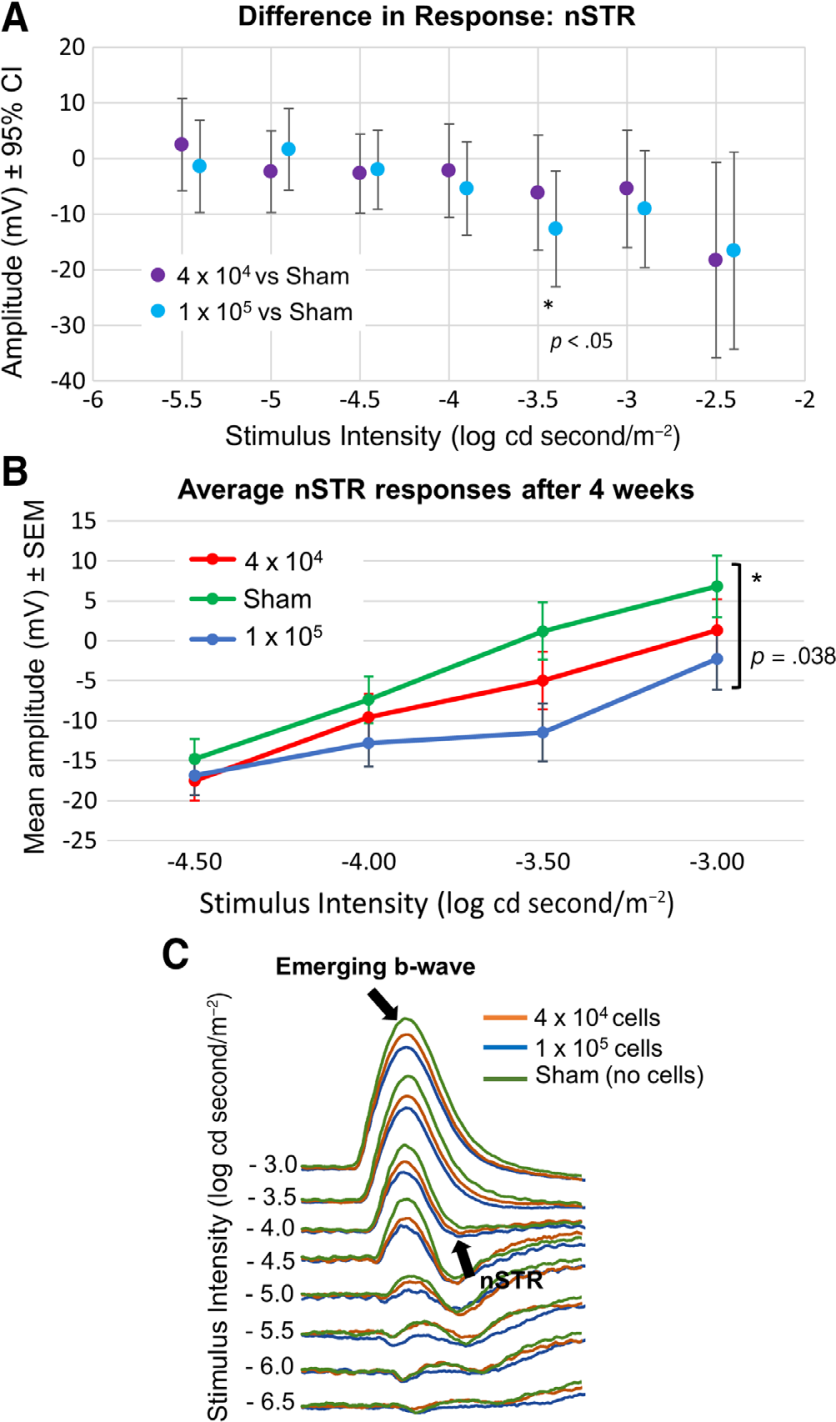
Electroretinogram (ERG) responses after cell transplantation in the NMDA damaged rat eye. **(A):** Differences in 95% CI of nSTR peak responses (measured between 160 and 230 millisecond) between human Müller glia and sham (medium only) treatments across all light intensities examined. **(B):** Comparison of the effects of human Müller glial cell transplantation with control treatment on the nSTR amplitude at 4 weeks postinjection across light intensities from −4.5 to −3 log cd seconds/m^2^ (*n* = 16 for each experimental group). **(C):** Scotopic ERG traces at −3 log to −6.5 log cd second/m^−2^ showing responses to medium (sham control, green lines), 1 × 10^5^ cells (blue lines) and 4 × 10^4^ cells (orange lines). Arrows indicate emerging b‐wave and nSTR. Abbreviations: CI, confidence interval; nSTR, negative scotopic threshold response.

Intravitreal cell transplantation was performed as previously described 15 following 2 weeks after intravitreal injection of NMDA to cause RGC damage. Sixteen animals were used in each of three experimental groups, receiving either a sham injection (medium alone), 4 × 10^4^ cells, or 1 × 10^5^ cells. To assess RGC function, scotopic ERGs were performed 4 weeks after cell transplantation to examine the nSTR, which denotes RGC function. Using 95% CI and compared with control treatment, statistical analysis of the effects of human Müller glia cell injection showed an increase in the amplitude of the nSTR across a range of relevant light intensities, with significant difference (*p* < .05) being demonstrated with 1 × 10^5^ human Müller glia cells at a light intensity of −3.5 log cd second/m^2^ (Fig. 5A). Furthermore, statistical analysis of the data obtained from eyes injected with both 4 × 10^4^ and 1 × 10^5^ iPSC‐derived human Müller glial cells when compared with control treatment demonstrated a dose response and significant improvement (*p* = .038) in the nSTR across light intensities ranging from −3.0 to −4.5 log cd second/m^2^ (Fig. 5B). Control animals which received NMDA treatment but no cell transplantation exhibited reduced nSTR and larger b‐waves for luminance −4 through to −3.0 log cd second/m^−2^ in comparison to animals receiving cell transplantations (Fig. 5C, arrows).

**Figure 6:**
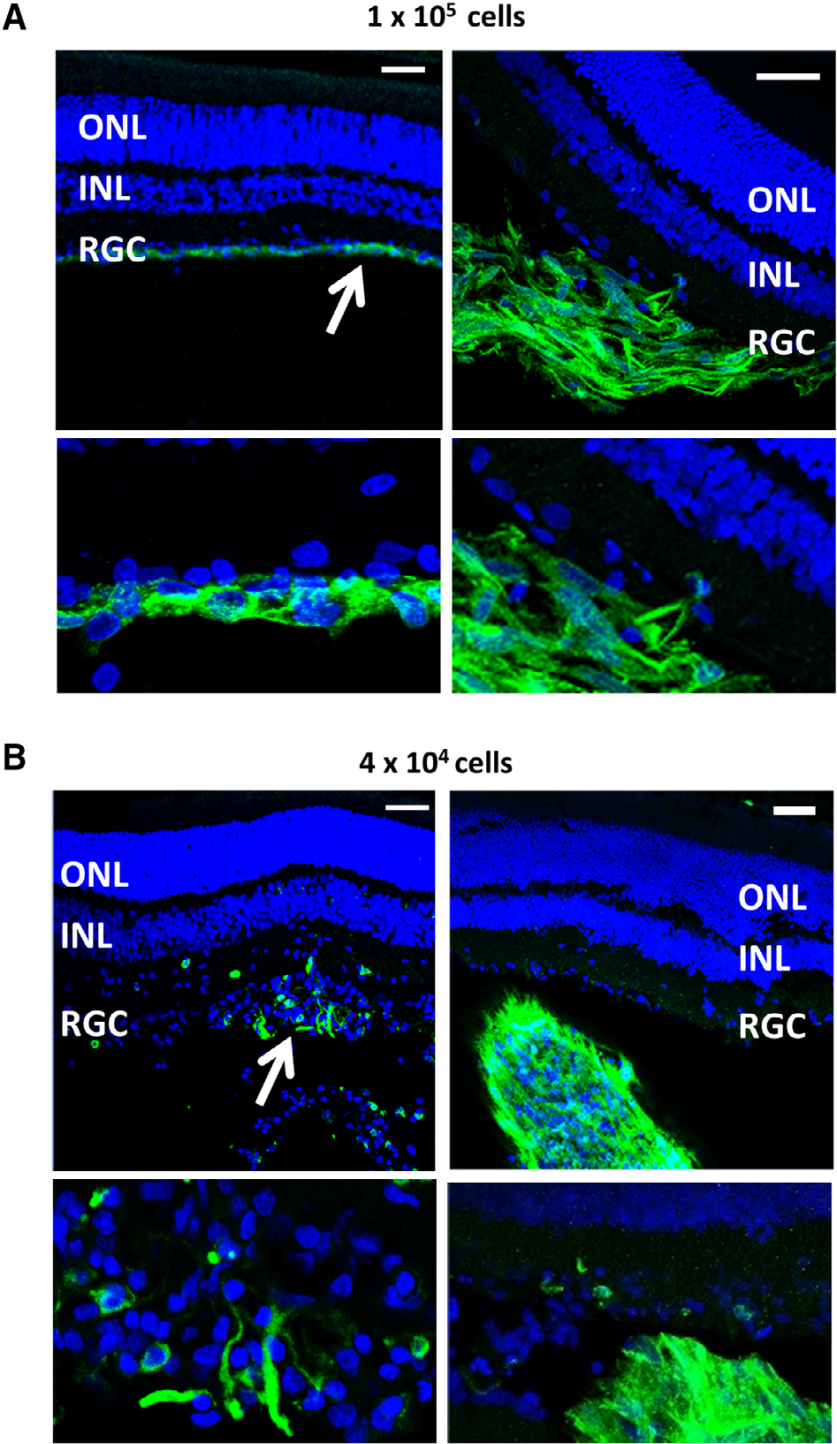
Localization of transplanted cells following immunostaining or retinal sections with antibodies to both human nestin plus CD29 and Alexa flour 488 used as a single secondary antibody. Images show eyes transplanted with **(A)** 1 × 10^5^ cells and **(B)** 4 × 10^4^ cells. White arrows indicate the location of the cells. Scale = 50 μm. Abbreviations: INL, inner nuclear layer; ONL, outer nuclear layer; RGC, retinal ganglion cell layer.

Antibodies to human CD29 and Nestin were used to locate the transplanted human cells in the rodent eye. Immunohistochemical staining of retinal sections from eyes transplanted with 1 × 10^5^ cells showed that cells were present in 13 out of the 16 eyes transplanted. Cells with healthy appearance were observed mostly in the vitreous (11 of 13), and although no integration of the transplanted Müller glia into the retina was observed, cells could be clearly seen strongly attached to the RGC layer in three cases (Fig. 6A, white arrow). In addition, transplanted cells were observed attached to the lens in two cases (not shown). Overall, fewer cells were observed in histological sections form eyes transplanted with 4 × 10^4^ cells as compared with those receiving the higher dose (illustrated on left image of Fig. 6B). In some cases, cell aggregates could be seen in the vitreous (illustrated on right image of Fig. 6B).

## Discussion

In this study, we have used human iPSC to produce retinal organoids for isolation and propagation of Müller glia in vitro. Müller cells isolated from the organoids were then used for transplantation into a rat model of RGC depletion by NMDA. Results presented in this study indicate that Müller glia that express CRALBP emerge at around day 90 after induction of differentiation of iPSC into retinal organoids. These observations are comparable to other studies that have reported expression of CRALBP in retinal organoids at similar stages of organoid formation 19. Upon transplantation, these cells partially restored RGC function in the NMDA‐damaged rat retina. These observations suggest that retinal organoids derived from iPSCs constitute an attractive source of Müller glia for cell transplantation therapies to treat retinal degenerative conditions. Therefore, we propose that Müller cells derived from organoids produced by pluripotent cells in vitro may be potentially used as a reliable and traceable source of cells for use in retinal therapies.

The data showed that Müller glia isolated from retinal organoids can be expanded over several passages and that their profile is comparable to published Müller cell lines derived from human cadaveric donors, including the established human MIO‐M1 Müller cell line 12, 13. Based on our previous studies, we have characterized these cells by their morphology as well as their gene and protein expression profiles which are distinctive of Müller glia. Isolated cells exhibited bipolar morphology and showed the ability to form sphere‐like structures that have been previously observed in the establishment of other Müller glia cell lines when cultured in the presence of FGF2 13. Cells isolated from the organoids expressed mRNA and protein coding for well‐known Müller glia markers including glutamine synthetase, CRALBP, nestin, and vimentin. The method of isolation is simple and exploited on the expression of CD29, a β1 integrin, by Müller glia, whose main ligand is fibronectin 22. Hence, adherence of Müller glia to fibronectin‐coated plates selectively promoted the emergence of highly pure populations of Müller glia (>97%) as determined by FACS analysis of the CD29 positive cell population, avoiding the need for further purification. This constitutes a simple method for selection and purification of Müller glia for regulatory compliance in the event that these cells can be used for cell therapies. It is also well documented that retinal neurons do not survive well in culture without specific on‐going support from extracellular signals 23, suggesting that only glia are likely to survive and proliferate in culture under the conditions used.

We also investigated the ability of Müller glia isolated from retinal organoids formed by iPSC to restore RGC function in rats depleted of RGC by NMDA as previously shown in our laboratory 15. This model mimics the pathological features of RGC damage observed in glaucoma and is a reliable model of RGC damage which can be easily assessed by measuring the nSTR response of the ERG 15. Following NMDA damage, we observed a reduction in the nSTR and a slight elevation in the b‐wave of the ERG, which is characteristic of similar glaucoma models 24. We observed a partial but significant restoration of the nSTR after transplantation of the highest dose of cells used (1 × 10^5^). Although injection of a smaller number of cells (4 × 10^4^) also resulted in an improvement in the nSTR, this response was not significant, indicating the need for a suitable number of cells for transplantation to improve retinal function. As with previous experimental models, we observed cell aggregates in a small number of animals (Fig. 6). However, we do not know whether this effect could interfere with visual function. We could speculate that because floaters present in human vitreous do not obstruct visual acuity, small cell aggregates may not interfere with vision. However, further studies are needed to assess the impact of these cell aggregates before any translation into the clinic.

## Conclusion

Results shown in this study are in agreement with previous findings from our laboratory, where we showed therapeutic benefit of the transplanted human Müller glia cell line MIO‐M1 in this rat model of glaucoma‐like damage 15. As previously observed in the Lister hooded rat and feline models of RGC depletion by NMDA 15, 16, cell integration into the retina was not observed, yet the RGC function was partially restored. Several studies have shown that RGC depletion occurs after NMDA injection, which at present requires ex vivo examination of the retinal tissue and prevents the assessment of transplantation in the same animal. Therefore, we undertook functional analysis by means of ERGs to assess RGC damage and the actual recovery of function in the NMDA‐treated animals. This also minimized the number of animals used for the study. Studies by Goldberg and collaborators have shown that if the soma of the RGC is still intact, the cell recovers function in response to neurotrophic factors, but if it is damaged, they will not be repaired by these factors 25. This is the mechanism by which we are proposing that Müller cells improve RGC function upon intravitreal transplantation.

Together the results show that Müller glia derived from pluripotent stem cells possess similar phenotypic characteristics and ability to partially restore visual function in NMDA depleted retina as Müller derived from the adult human retina. On this basis, it can be suggested that iPSC‐derived Müller glia may constitute a valuable resource for transplantation studies that could be potentially translated into the clinic.

## Author contributions

G.A.L.: conception/design, financial support, manuscript writing, final approval of manuscript; K.E.: conception/design, collection and/or assembly of data, data analysis and interpretation, manuscript writing; W.W., C.M.‐D., A.V., M.S.: collection and/or assembly of data; H.J., P.C.: provision of study materials; A.J.F.C., C.M.R.: administrative support, provision of study materials; K.G.: data analysis and interpretation; N.C.: conception/design, financial support, administrative support, provision of study materials final approval of manuscript; P.T.K.: conception/design, final approval of manuscript.

## Disclosure of Potential Conflicts of Interest

H.J. declared consultant/advisory role with Allergan and honoraria from Santen, Allergan, and Laboratories Thea. K.G. declared consultant/advisory role with Apollo Therapeutics. N.C. declare employment with Apollo Therapeutics that funded research in Prof. Limb's lab. P.T.K. declare inventor or patent holder on Müller Cells and the use in retinal regeneration, consultant/advisory role and received honoraria from Santen, Belkin, Novartis, and Aerie, and received research funding from Apollo Therapeutics. G.A.L. declared inventor or patent holder on Müller cells and their use in retinal regeneration. The other authors indicated no potential conflicts of interest.

## Supporting information


**Supporting Information Figure S1 Flow cytometry analysis of negative Müller glia markers.** Flow cytometry analysis of Müller glia isolated from organoids showing cells were (A) 0.7% positive for the epithelial marker cytokeratin‐18 and (B) 0.2% positive for the stem cell marker SSEA‐4.

**Supporting Information Figure S2 Flow cytometry analysis of isotype control antibodies.** Flow cytometry analysis of Müller glia isolated from organoids showing isotype controls for antibodies to (A) CD44 and CD29 (B) Cytokeratin‐18 (C) SSEA‐4 were less than 2% positive in the cell preparation.

**Supporting Information Figure S3 Assessment of RGC function follwoing intravitreal NMDA injections.** Box‐plot shows the average nSTR amplitude at −3.5 log cd s m^−2^ for the NMDA treated eye in comparison to the untreated fellow eye at 1 week after NMDA injection, n = 5 (p = .0302; students *t*‐test).

**Supplementary Table 1**. List of Antibodies used in the study
**Supplementary Table 2**. List of primers used in the study.

## References

[1] Pascolini D , Mariotti SP . Global estimates of visual impairment: 2010. Br J Ophthalmol 2012;96:614–618. 22133988 10.1136/bjophthalmol-2011-300539

[2] Diekmann H , Fischer D . Glaucoma and optic nerve repair. Cell Tissue Res 2013, 353, 327, 337. 23512141 10.1007/s00441-013-1596-8

[3] Quigley HA . Neuronal death in glaucoma. Prog Retin Eye Res 1999;18:39–57. 9920498 10.1016/s1350-9462(98)00014-7

[4] Raymond P , Barthel L , Bernardos R , Perkowski J. Molecular characterization of retinal stem cells and their niches in adult zebrafish. BMC Dev Biol 2006;6, 36. 16872490 10.1186/1471-213X-6-36 PMC1564002

[5] Nelson CM , Hyde DR . Müller glia as a source of neuronal progenitor cells to regenerate the damaged zebrafish retina. In: MM LaVail, Ash JD, Anderson RE, Hollyfield JG, Grimm C, eds. Retinal Degenerative Diseases. Vol 723 Springer, 2012:425–430. 10.1007/978-1-4614-0631-0_54 22183361

[6] Thummel R , Kassen SC , Enright JM , Nelson CM , Montgomery JE , Hyde DR . Characterization of Müller glia and neuronal progenitors during adult zebrafish retinal regeneration. Exp Eye Res 2008;87:433–444. 18718467 10.1016/j.exer.2008.07.009 PMC2586672

[7] Lenkowski JR , Raymond PA . Müller glia: Stem cells for generation and regeneration of retinal neurons in teleost fish. Prog Retin Eye Res 2014, 40, 94, 123. 24412518 10.1016/j.preteyeres.2013.12.007 PMC3999222

[8] Hagerman GF , Noel NC , Cao SY , DuVal MG , Oel AP , Allison WT . Rapid recovery of visual function associated with blue cone ablation in zebrafish. PLoS One 2016;11:e0166932. 27893779 10.1371/journal.pone.0166932 PMC5125653

[9] Fischer AJ , Reh TA . Muller glia are a potential source of neural regeneration in the postnatal chicken retina. Nat Neurosci 2001;4:247–252. 11224540 10.1038/85090

[10] Karl MO , Hayes S , Nelson BR , Tan K , Buckingham B , Reh TA . Stimulation of neural regeneration in the mouse retina. Proc Natl Acad Sci USA 2008;105:19508–19513. 19033471 10.1073/pnas.0807453105 PMC2614791

[11] Ooto S , Akagi T , Kageyama R , Akita J , Mandai M , Honda Y , Takahashi M Potential for neural regeneration after neurotoxic injury in the adult mammalian retina. Proc Natl Acad Sci USA 2004;101:13654–13659. 15353594 10.1073/pnas.0402129101 PMC518808

[12] Limb GA , Salt TE , Munro PMG , Moss SE , Khaw PT . In vitro characterization of a spontaneously immortalized human müller cell line (MIO‐M1). Invest Ophthalmol Vis Sci. 2002;43:864–869. 11867609

[13] Lawrence JM , Singhal S , Bhatia B , Keegan DJ , Reh TA , Luthert PJ , Khaw PT , Limb GA MIO‐M1 cells and similar Müller glial cell lines derived from adult human retina exhibit neural stem cell characteristics. Stem Cells 2007;25:2033–2043. 17525239 10.1634/stemcells.2006-0724

[14] Becker S , Singhal S , Jones MF , Eastlake K , Cottrill PB , Jayaram H , Limb GA Acquisition of RGC phenotype in human Muller glia with stem cell characteristics is accompanied by upregulation of functional nicotinic acetylcholine receptors. Mol Vis 2013;19:1925–1936. 24049438 PMC3774575

[15] Singhal S , Bhatia B , Jayaram H , Becker S , Jones MF , Cottrill PB , Khaw PT , Salt TE , Limb GA Human Müller glia with stem cell characteristics differentiate into retinal ganglion cell (RGC) precursors in vitro and partially restore RGC function in vivo following transplantation. Stem Cells Translational Medicine 2012;1:188–199. 23197778 10.5966/sctm.2011-0005 PMC3659849

[16] Becker S , Eastlake K , Jayaram H , Jones MF , Brown RA , McLellan GJ , Charteris DG , Khaw PT , Limb GA Allogeneic transplantation of muller‐derived retinal ganglion cells improves retinal function in a feline model of ganglion cell depletion. Stem Cells Translational Medicine 2016;5:192–205. 26718648 10.5966/sctm.2015-0125 PMC4729554

[17] Jayaram H , Jones MF , Eastlake K , et al. Transplantation of photoreceptors derived from human Muller glia restore rod function in the P23H rat. Stem Cells Translational Medicine 2014:323–333. 24477073 10.5966/sctm.2013-0112 PMC3952927

[18] Nakano T , Ando S , Takata N , Kawada M , Muguruma K , Sekiguchi K , Saito K , Yonemura S , Eiraku M , Sasai Y Self‐formation of optic cups and storable stratified neural retina from human ESCs. Cell Stem Cell 2012;10:771–785. 22704518 10.1016/j.stem.2012.05.009

[19] Zhong X , Gutierrez C , Xue T , Hampton C , Vergara MN , Cao LH , Peters A , Park TS , Zambidis ET , Meyer JS , Gamm DM , Yau KW , Canto‐Soler MV Generation of three‐dimensional retinal tissue with functional photoreceptors from human iPSCs. Nat Commun 2014;5:4047. 24915161 10.1038/ncomms5047 PMC4370190

[20] Carter DA , Smart MJ , Letton WV , et al. Mislocalisation of BEST1 in iPSC‐derived retinal pigment epithelial cells from a family with autosomal dominant vitreoretinochoroidopathy (ADVIRC). Sci Rep 2016;6:33792. 27653836 10.1038/srep33792 PMC5031956

[21] Parfitt DA , Lane A , Ramsden C , Jovanovic K , Coffey PJ , Hardcastle AJ , Cheetham ME Using induced pluripotent stem cells to understand retinal ciliopathy disease mechanisms and develop therapies. Biochem Soc Trans 2016;44:1245–1251. 27911706 10.1042/BST20160156 PMC5238943

[22] Matsuyama T , Yamada A , Kay J , Yamada KM , Akiyama SK , Schlossman SF , Morimoto C Activation of CD4 cells by fibronectin and anti‐CD3 antibody. A synergistic effect mediated by the VLA‐5 fibronectin receptor complex. J Exp Med 1989;170:1133–1148. 2477485 10.1084/jem.170.4.1133 PMC2189458

[23] Meyer‐Franke A , Kaplan MR , Pfrieger FW , Barres BA . Characterization of the signaling interactions that promote the survival and growth of developing retinal ganglion cells in culture. Neuron 1995;15:805–819. 7576630 10.1016/0896-6273(95)90172-8

[24] Khan AK , Tse DY , van der Heijden ME , Shah P , Nusbaum DM , Yang Z , Wu SM , Frankfort BJ Prolonged elevation of intraocular pressure results in retinal ganglion cell loss and abnormal retinal function in mice. Exp Eye Res 2015;130:29–37. 25450059 10.1016/j.exer.2014.11.007 PMC4308057

[25] Goldberg JL , Espinosa JS , Xu Y , Davidson N , Kovacs GT , Barres BA . Retinal ganglion cells do not extend axons by default: Promotion by neurotrophic signaling and electrical activity. Neuron 2002;33:689–702. 11879647 10.1016/s0896-6273(02)00602-5

