# Supplementary figures and images for: Phenotypic and Functional Characterization of Müller Glia Isolated from Induced Pluripotent Stem Cell‐Derived Retinal Organoids: Improvement of Retinal Ganglion Cell Function upon Transplantation
Source: Stem Cells Transl Med. 2019 Apr 29;8(8):775–84. doi: 10.1002/sctm.18-0263 (PMC6646702; doi:10.1002/sctm.18-0263)

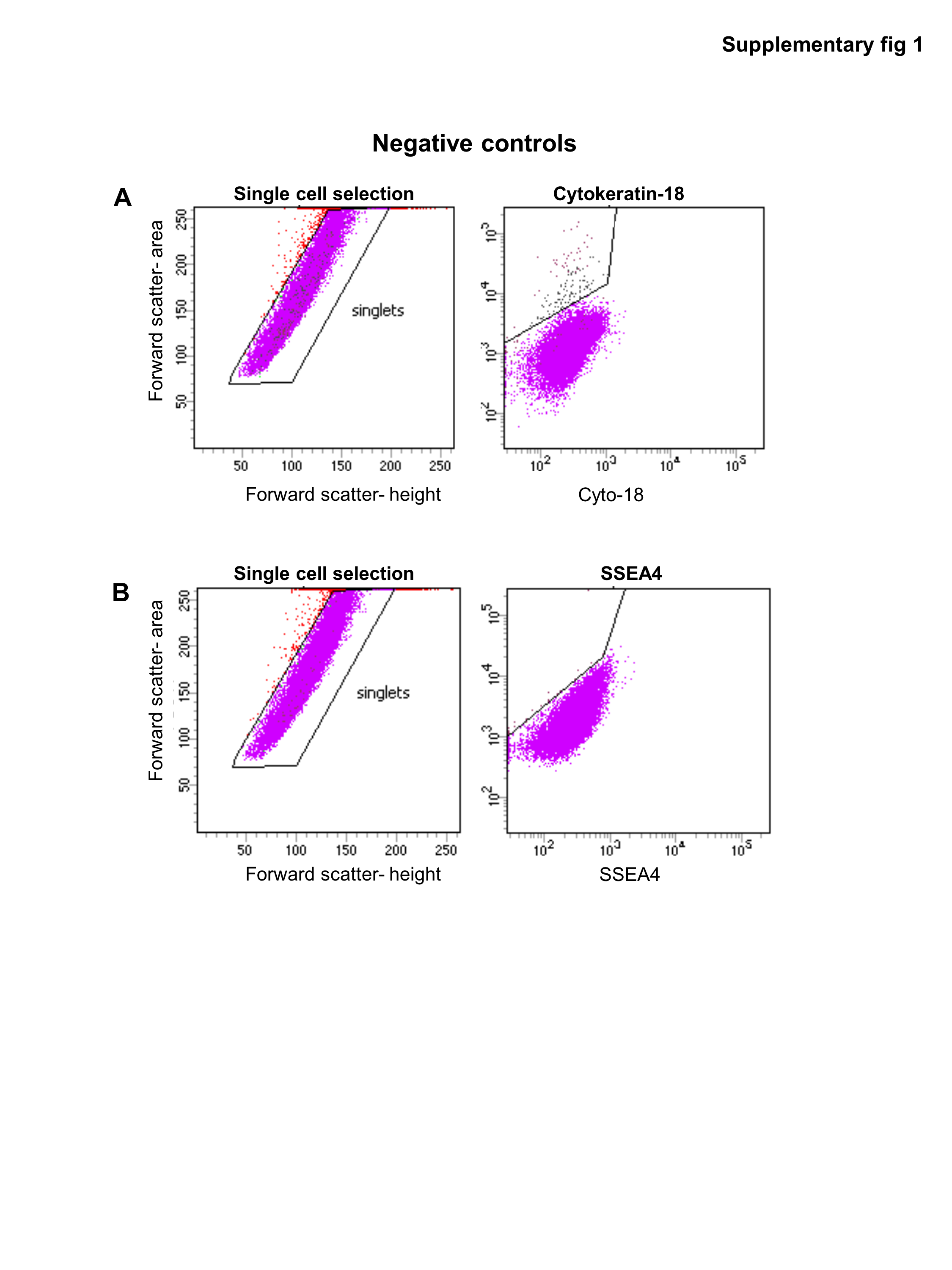

Supplement: Supplementary file 1 — Supporting Information Figure S1 Flow cytometry analysis of negative Müller glia markers. Flow cytometry analysis of Müller glia isolated from organoids showing cells were (A) 0.7% positive for the epithelial marker cytokeratin‐18 and (B) 0.2% positive for the stem cell marker SSEA‐4. [file SCT3-8-775-s001.TIF]

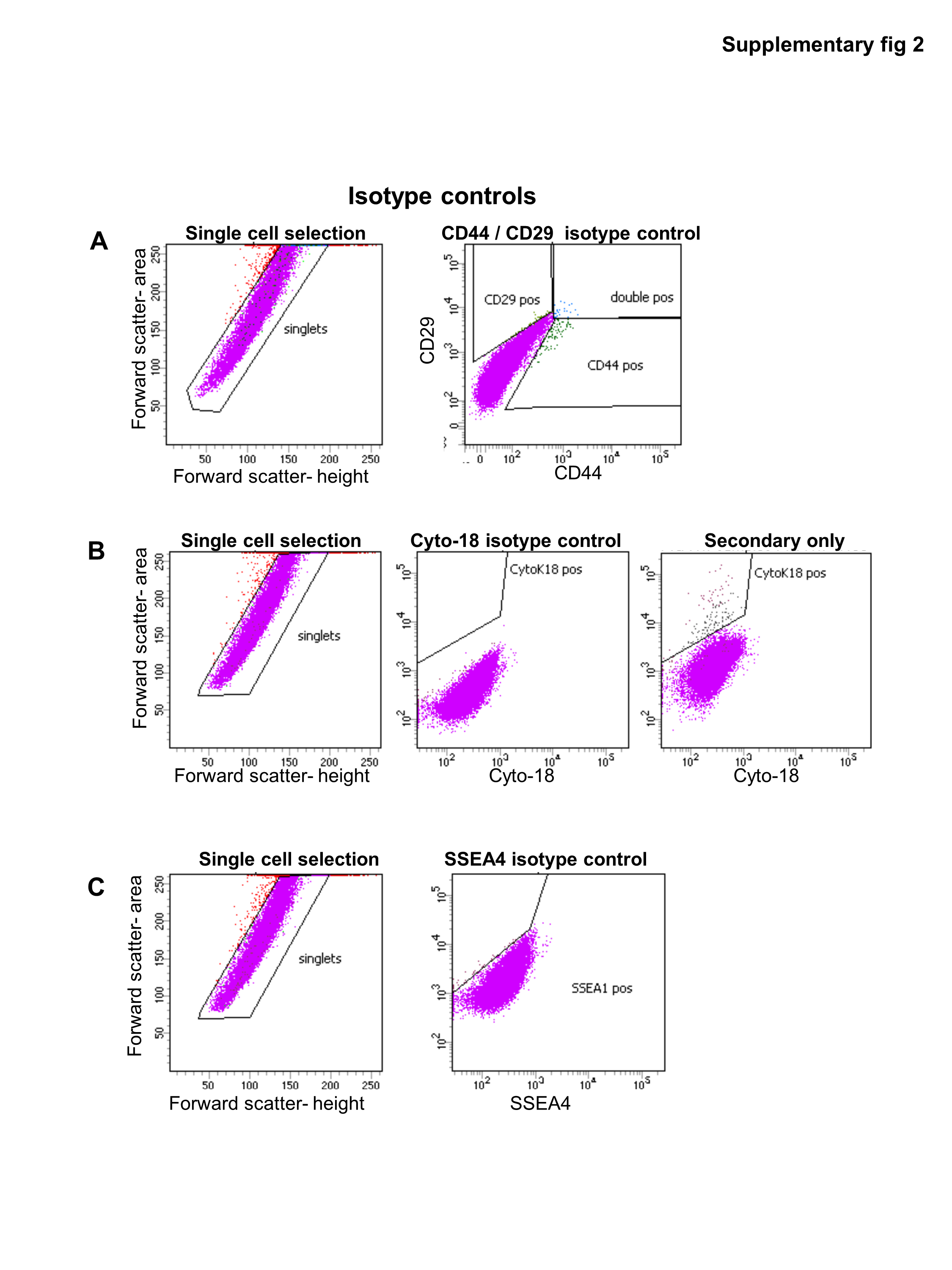

Supplement: Supplementary file 2 — Supporting Information Figure S2 Flow cytometry analysis of isotype control antibodies. Flow cytometry analysis of Müller glia isolated from organoids showing isotype controls for antibodies to (A) CD44 and CD29 (B) Cytokeratin‐18 (C) SSEA‐4 were less than 2% positive in the cell preparation. [file SCT3-8-775-s002.TIF]

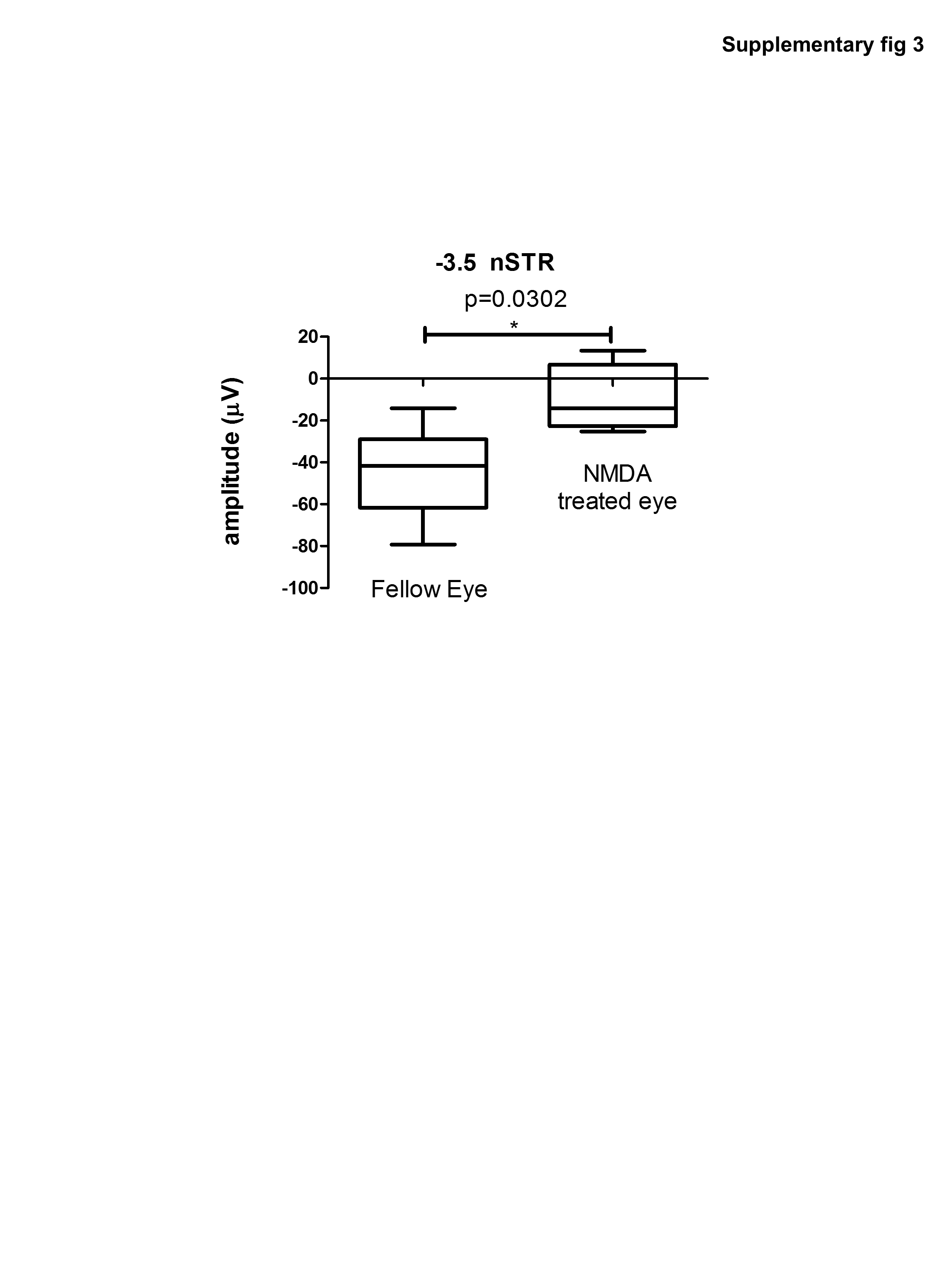

Supplement: Supplementary file 3 — Supporting Information Figure S3 Assessment of RGC function follwoing intravitreal NMDA injections. Box‐plot shows the average nSTR amplitude at −3.5 log cd s m−2 for the NMDA treated eye in comparison to the untreated fellow eye at 1 week after NMDA injection, n = 5 (p = .0302; students t‐test). [file SCT3-8-775-s003.TIF]
