# Supplementary material for: Phenotypic and Functional Characterization of Müller Glia Isolated from Induced Pluripotent Stem Cell‐Derived Retinal Organoids: Improvement of Retinal Ganglion Cell Function upon Transplantation
Source: Stem Cells Transl Med. 2019 Apr 29;8(8):775–84. doi: 10.1002/sctm.18-0263 (PMC6646702; doi:10.1002/sctm.18-0263)
Supplement: Supplementary file 4 — Supplementary Table 1. List of Antibodies used in the study Supplementary Table 2. List of primers used in the study. [file SCT3-8-775-s004.docx]

**Supplementary Table 1. List of Antibodies used in the study**

| ***Antibody used for IHC*** | ***Supplier / Cat. No.*** | ***Host*** | ***Dilution*** |
| --- | --- | --- | --- |
| CD29 | Santa Cruz – sc59829 | mouse | 1:300 |
| CD44 | Abcam – ab157107 | rabbit | 1:500 |
| Glutamine synthetase | Novus biologicals – NB110-41404 | rabbit | 1:500 |
| CRALBP | Novus Biologicals – NB100-74392 | mouse | 1:50 |
| Vimentin | Santa Cruz – sc5565 | rabbit | 1:100 |
| Nestin | eBiosciences – 14-9843-80 | mouse | 1:250 |
| Sox9 | Santa Cruz – sc20095 | Rabbit | 1:200 |
| ***Antibodies used for FACs*** | ***Supplier*** | ***Host*** | ***Dilution*** |
| CD29 REA1060 VioBr515 | Miltenyi Biotech -130-118-194 | - | 1:5 |
| CD44 REA690 APC | Miltenyi Biotech – 130-113-900 | - | 1:5 |
| SSEA-4 REA232 VioGreen | Miltenyi Biotech- 130-113-338 | - | 1:2 |
| Cytokeratin-18 | Abcam – Ab32118 | Rabbit | 1:20 |
| Donkey anti-rabbit IgG BV241 (Secondary antibody for cytokeratin-18) | Biolegend- 406410 |  | 10ul Neat |
| ***Primary antibody isotype controls*** | ***Supplier*** | ***Host*** | ***Dilution*** |
| REA293 VioBr515 | Miltenyi Biotech -130-113-445 |  | 1:5 |
| REA-APC | Miltenyi Biotech- 130-113-434 |  | 1:5 |
| REA – VioGreen | Miltenyi Biotech- 130-113-444 |  | 1:2 |
| Rb IgG dilued to 17ug/ml | Abcam – ab172730 |  | 1:20 |

**Supplementary Table 2. List of primers used in the study.**

| **Gene** | **Primer Sequence**  **(F forward; R reverse)** | **Product size (base pairs: bp)** | **Annealing temperature** | **Number of cycles** |
| --- | --- | --- | --- | --- |
| Beta Actin | F-CATGTACGTTGCTATCCAGGC  R-CTCCTTAATGTCACGCACGAT | 250 | 54 | 26 |
| Glutamine synthetase | F- TGGGAGCAGACAGAGCCTAT  R- CAGGAATGGGCTTAGGATCA | 240 | 60 | 28 |
| CRALBP | F- AGGCTGGGAGTTTTCCACAC  R- TCTCCCAGACAGATGGAGGG | 170 | 60 | 38 |
| Vimentin | F- GAGAACTTTGCCGTTGAAGC  R- TCCAGCAGCTTCCTGTAGGT | 170 | 58 | 28 |
| GFAP | F- CTGGGCTCAAGCAGTCTACC  R-GAGTCATCGCTCAGGAGGTC | 187 | 58 | 36 |
| Nestin | F-CTGCTACCCTTGAGACACCTC  R-GGGCTCTGATCTCTGCATCTAC | 141 | 60 | 30 |
| Notch | F- GGAGGCATCCTACCCTTTTC  R- TGTGTTGCTGGAGCATCTTC | 118 | 60 | 28 |
| Sox2 | F- GGCAGCTACAGCATGATGC  R- TCGGACTTGACCACCGAAC | 236 | 60 | 30 |
| Pax6 | F- AGATGAGGCTCAAATGCGAC  R- GTTGGTAGACACTGGTGCTG | 300 | 60 | 30 |
| Chx10 | F- AGCAGAGGAGCTGGAGAAG  R-CATGATGCCATCCTTGGCTG | 258 | 60 | 36 |
| Sox9 | F-ACGACTACACCGACCACCA  R-TAGGATCATCTCGGCCATC | 256 | 60 | 26 |
